# Association Study of Polymorphisms in Selenoprotein Genes and Kashin–Beck Disease and Serum Selenium/Iodine Concentration in a Tibetan Population

**DOI:** 10.1371/journal.pone.0071411

**Published:** 2013-08-23

**Authors:** Lulin Huang, Yi Shi, Fang Lu, Hong Zheng, Xiaoqi Liu, Bo Gong, Jiyun Yang, Ying Lin, Jing Cheng, Shi Ma, He Lin, Zhenglin Yang

**Affiliations:** 1 Center for Human Molecular Biology & Genetics, The Institute of Laboratory Medicine, Sichuan Academy of Medical Sciences & Sichuan Provincial People's Hospital, Chengdu, Sichuan, China; 2 Sichuan Translational Research Hospital, Chinese Academy of Sciences, Chengdu, Sichuan, China; Vanderbilt University, United State of America

## Abstract

**Background:**

Kashin-Beck disease is a kind of degenerative osteoarthropathy. Genetic factors may play an important role in the pathogenesis of KBD.

**Objective:**

To investigate the association of the selenoprotein genes *GPX1* (rs1050450, rs1800668, and rs3811699), *TrxR2* (rs5748469), and *DIO2* (rs225014) with Kashin-Beck disease (KBD) in a Tibetan population and to investigate the association of these SNPs with the serum iodine/selenium concentration in the Tibetan population.

**Design:**

Five SNPs including rs1050450, rs1800668, and rs3811699 in the *GPX1* gene, rs5748469 in the *TrxR2* gene, and rs225014 in the *DIO2* gene were analyzed in Tibetan KBD patients and controls using the SNaPshot method. *P* trend values of the SNPs were calculated using an additive model.

**Results:**

None of the five SNPs in the three genes showed a significant association with KBD. Haplotypes TCC, TTC and TTT of rs1050450, rs1800668 and rs3811699 in *GPX1* showed a significant association with KBD and controls with *P* value of 0.0421, 5.0E-4 and 0.0066, respectively. The *GPX1* gene (rs1050450) showed a potential significant association with the iodine concentration in the Tibetan study population (*P* = 0.02726). However, no such association was detected with the selenium concentration (*P* = 0.2849).

**Conclusion(s):**

In this study, we showed that single SNPs in the genes *GPX1* (rs1050450, rs1800668 and rs3811699), *TrxR2* (rs5748469), and *DIO2* (rs225014) may not be significantly associated with KBD in a Tibetan population. However, haplotype analysis of SNPs rs1050450, rs1800668 and rs3811699 in *GPX1* gene showed a significant association with KBD. The results suggested that *GPX1* gene play a protective role in the susceptivity of KBD in Tibetans. Furthermore, the *GPX1* gene (rs1050450) may be significantly associated with the serum iodine concentration in Tibetans.

## Introduction

Kashin–Beck Disease (KBD) is named after the two Russian Cossack doctors Nikolai Kashin and Evgeny Beck who first described bone deformities in patients in Russia in 1848 and 1906, respectively [1]. Today, KBD is known as an endemic, chronic, and degenerative osteoarthropathy, with the involvement of epiphyseal cartilage damage, joint damage, and gradual deformation of the bone and joints [2]–[4]. KBD is endemic in a crescent-shaped area encompassing South-Eastern Siberia to North China, Central China, and Chinese Tibet; it is also endemic in Mongolia and North Korea [5]. China has the most KBD patients in the world [1]. The most frequently involved joints are the ankles, knees, wrists, and elbows. The disease often occurs in children aged 5–15 years and is age related, and serious KBD is responsible for significant disability. In some KBD endemic regions in China, the incidence of KBD is about 8.3% (2.5 million of 30 million urban residents affected) [6].

The etiology of KBD is largely unknown. The risk factors are thought to include deficiency in trace elements, mainly selenium and iodine deficiency [7]–[14]. In addition, mycotoxins such as Trichothecene mycotoxin (T-2), which are produced by various fungi such as *F. compactum*, *F. moniliforme*, and *F. oxysporum* in contaminated storage grains, are also suspected factors in KBD susceptibility [15]–[17]. Organic substances such as humic acid and fulvic acid in drinking water have also been implicated in the disease [3], [18], [19]. Among all of these risk factors, selenium and iodine have been extensively studied. Recently we also confirmed that low selenium and iodine concentrations are associated with KBD [20].

Genetic factors also play an important role in the pathogenesis of KBD. Xiong *et al*. showed that the polymorphisms of the selenoprotein *GPX1* gene (rs1050450 and Pro200Leu) were significantly different between patients with KBD and controls (*P* = 0.013) in a Han Chinese population [21]. To further investigate the potential relationship between selenoprotein genes and KBD susceptibility in Tibetans, we analyzed the association of the three selenoprotein genes *GPX1* (rs1050450, rs1800668, and rs3811699), *TrxR2* (rs5748469), and *DIO2* (rs225014) with Tibetan KBD in this study. Moreover, we investigated the association between these SNPs and serum selenium and iodine concentrations in Tibetans.

## Materials and Methods

### Study population

**Figure 1:**
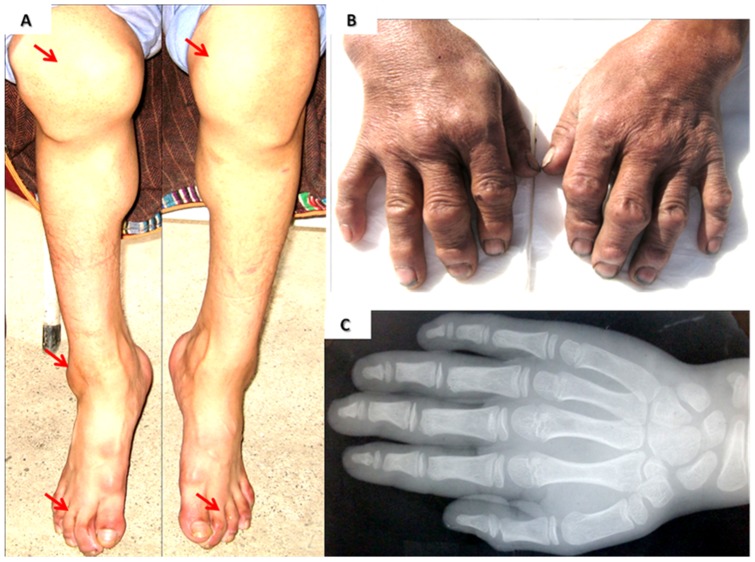
Clinical features of KBD patients. A. A female KBD patient with her knee, ankle and toe joints deformity, the arrows show multiple joints of this patient are affected. B. A male KBD patient with his fingers joints deformity. C. The X-ray picture of a KBD patient's hand.

KBD patients and matched normal controls in this study were recruited from Tibetan populations in the same endemic villages in Song Pan, Ruo Er Gai, and Hong Yuan counties in the Aba Tibetan Autonomous prefecture of Sichuan Province, China. Clinical examination was performed as the methods of Moreno-Reyes [7] and Greulich [22]. Patients show joint of fingers, toes, knees and ankles swelling (Figure 1). The KBD patients showed specific changes on X-ray photography; they did not have other arthritis diseases such as rheumatoid arthritis (RA), Osteoarthritis (OA), or local inflammation. Normal controls were individuals with a normal joint examination and no other bone or joint disease. Clinical information about the patients and the controls is listed in Table 1. Veinal bloods (5 ml) of the participants were collected for DNA extraction. The Institutional Review Boards of the Sichuan Academy of Medical Sciences & Sichuan Provincial People's Hospital approved this study. All of the participants received and signed the informed consent.

**Table 1:** Characteristics of the KBD cases and controls.

Characteristics	Cases(n = 638)	Controls(n = 324)
Age, mean ± SD	53.3±13.07	54.5±15.81
Sex, male/female	244/394	157/167
Degree		
I	241	–
II	336	–
III	60	–
IV	1	–

### Selection of SNPs

Following a review of the literature, we selected five single nucleotide polymorphisms (SNPs) in three selenoprotein genes to genotype: *glutathione peroxidase 1* (*GPX1*, rs1050450), thioredoxin reductase 2 (*TrxR2*, rs5748469), and iodothyronine deiodinase type 2 (*DIO2*, rs225014) to genotype [21]. We also referenced the NCBI SNP database and selected rs1800668 and rs3811699 in the *GPX1* gene for genotyping. Information pertaining to the five SNPs selected is shown in Table 2.

**Table 2:** Conditions used for genotyping assays.

SNPs	Primers	Ta(°C)
GPX1(rs1050450)	FW:CGCCAAGAACGAAGAGATTC	58
missense CCC⇒ CTC Pro200Leu	RV:CTGACACCCGGCACTTTATT	
	S30-C/T:CTGACATCGAAGCCCTGCTGTCTCAAGGGC	
	SV40-A/G:CCAAGCAGCCGGGGTAGGAGGGGCGCCCTAGGCACAGCTG	
GPX1(rs1800668)	FW:ACAGGAGAGGAGGGCTGTTT	63.5
UTR-5 C/T	RV:AGAAGGCATACACCGACTGG	
	S60-C/T:CCTGTGCCACGTGACCCGCCGCCGGCCAGTTAAAAGGAGGCGCCTGCTGGCCTCCCCTTA	
GPX1(rs3811699)	FW:AGGACTTCCTGGCCTAGCTC	63.3
nearGene-5 C/T	RV:CCAGAGGGATCTAGGCTTCC	
	S50C/T:GCCAAGGAAACGCTGCCGGAGTCCTCCCTCCCTGGCCTCCTCAGGCTGCA	
DIO2(rs225014)	FW:TCGTGAAAGGAGGTCAAGT	58
missense C/T ACA⇒ GCA Thr92Ala;Thr128Ala	RV:GTGGCAATGTGTTTAATGTG	
	S50-C/T:CTCAGCTATCTTCTCCTGGGTACCATTGCCACTGTTGTCACCTCCTTCTG	
TrxR2(rs5748469)	FW:TCCCAAAGTGCTGTGAGT	58
missense A/C GCC⇒ TCC Ala66Ser	RV:ACCTGCTGCTCTCCTTATC	
	S40-A/C:TGCCTACCTTGGGGAGAAGGTTCCACGTAGTCCACCACGG	

### Genotyping

Genomic DNA was extracted using a Gentra Puregene Blood DNA kit (Minneapolis, MN). SNP genotyping was performed by the dye terminator-based SNaPshot method (Applied Biosystems, Foster City, CA). The SNP analysis was performed on the ABI 3130XL genetic analyzer (Applied Biosystems). The genotypes of the SNPs were determined by Genemapper software (Applied Biosystems). All of the SNPs reported in this manuscript had a genotyping success rate >96 percent and accuracy as judged by random regenotyping of 10 percent of the samples in the subject group. The PCR and SNaPshot primers are listed in Table 2.

### Statistical analysis

The Hardy-Weinberg equilibrium (HWE) for each SNP polymorphism was tested by the χ2 test with df  = 1. The *P* values of the SNPs were calculated using an additive model. Unadjusted odds ratios of the alleles and genotypes were estimated by the χ2 test. The LD block structure was examined by using software Haploview Vision 4.2. The D and r2 values for all pairs of SNPs were calculated, and the haplotype blocks were estimated by using software Haploview Vision 4.2.All of the statistical analyses were performed using the software SPSS version 10.0. *P*<0.05 was considered statistically significant.

## Results

### Genotype analysis of the KBD group and the controls

All of the five SNPs selected were successfully genotyped. However, the results showed that none of these five SNPs were significantly associated with KBD (Table 3). Although the SNP rs1050450 (Pro200Leu) in the *GPX1* gene was reported to be significantly associated with KBD in previous studies in a Han Chinese population (*P* = 0.013), this SNP did not show a significant association with KBD in the Tibetan population in this study (*P* =  0.1031) [21]. The other two SNPs in the *GPX1* gene (rs1800668 at 5′UTR of the gene and rs3811699 at 3′upstream of the gene) also showed no association with KBD (*P* = 0.7614 and *P* = 0.8351, respectively). In contrast to the results that Xiong *et al*. reported in the Han population, neither the *DIO2* gene (rs225014) nor the *TrxR2* gene (rs5748469) showed significant differences in the Tibetan KBD group compared with the control group (*P* = 0.7287 and *P* = 0.4426, respectively).

**Table 3:** Genotype and allele frequencies of polymorphisms across selenoprotein genes in Tibetan.

SNPs	Controls		KBD		Trend
	number	Freq	number	Freq.	p-value(OR)
GPX1(rs1050450)					0.1031(0.74)
CC	271	0.836	559	0.876	
CT	53	0.164	79	0.124	
TT	0	0	0	0	
C-allele	595	0.918	1197	0.938	
T-allele	53	0.082	7	0.062	
*P_HWE*	0.1089		0.0956		
GPX1(rs1800668)					0.7614(1.06)
CC	183	0.806	197	0.804	
CT	40	0.176	41	0.167	
TT	4	0.018	7	0.029	
C-allele	406	0.894	435	0.883	
T-allele	48	0.106	55	0.112	
*P_HWE*	0.3046		0.012		
GPX1(rs3811699)					0.8351(0.95)
TT	202	0.838	212	0.841	
CT	35	0.145	37	0.147	
CC	4	0.017	3	0.012	
T-allele	439	0.911	461	0.915	
C-allele	43	0.089	43	0.085	
*P_HWE*	0.0988		0.3467		
DIO2(rs225014)					0.7287(1.04)
TT	94	0.388	158	0.356	
CT	113	0.467	228	0.514	
CC	35	0.145	58	0.131	
T-allele	301	0.622	544	0.613	
C-allele	183	0.378	344	0.387	
*P_HWE*	0.9121		0.0844		
TrxR2(rs5748469)					0.4426(0.86)
AA	137	0.714	209	0.749	
AC	50	0.26	63	0.226	
CC	5	0.026	7	0.025	
A-allele	324	0.844	481	0.882	
C-allele	60	0.156	77	0.138	
*P_HWE*	0.8642		0.3958		

### Haplotype analysis of the three SNPs in the *GPX1* gene

We examined the three SNPs in gene *GPX1* (rs3811699, rs1050450, and rs1800668) in all tested samples using the program Haploview (Vision4.2). The haplotypes TCC, TTC and TTT generated from these three SNPs proved to be significantly different between the KBD cases and controls (*P* = 0.0421, 0.0005 and 0.0066, Table 4). These individuals showed protective feature in the susceptibility of KBD with the odds ratios of 0.69, 0.22 and 0.15 respectively.

**Table 4:** The haplotype association of *gpx1* with KBD in this study.

Haplotype	Frequencies	Chi Square	P value	Odds ratio (95% CI)
TCC	0.848	4.132	0.0421	0.69(0.49–0.98)
CTT	0.06	0.222	0.6376	1.14(0.67–1.91)
TTC	0.032	12.183	5.00E-04	0.22(0.088–0.553)
CCT	0.022	0.436	0.5092	1.34(0.57–3.14)
TCT	0.016	0.296	0.5861	1.32(0.49–3.57)
TTT	0.015	7.371	0.0066	0.15(0.03–0.724)

### Comparison of the Results of the Genotype Analysis Based on the Subjects' Iodine or Selenium Concentrations

Given that KBD is related to iodine or selenium deficiency, we analyzed the genotypes of the KBD group and the controls in relation to the subjects' serum iodine or selenium concentrations which were tested previously [23]. The results are shown in Table 5 and Table 6 for iodine and selenium, respectively. We found no significant differences between the KBD group and the controls, providing additional evidence for the absence of an association between the five SNPs and KBD in the Tibetan population. The power calculation results are shown in Table 7.

**Table 5:** Genotype and allele frequencies of polymorphisms across selenoprotein genes by serum iodine concentration in Tibetan population.

SNPs	Controls	KBD	Controls	KBD	Trend	Higher group	Lower group	Higher group	Lower group	Trend
	number	number	Freq.	Freq.	p-value	number	number	Freq.	Freq.	p-value
GPX1(rs1050450)					0.9129					*0.02726
CC	146	155	0.811	0.816		154	147	0.77	0.865	
CT	34	35	0.189	0.184		46	23	0.23	0.135	
TT	0	0	0	0		0	0	0	0	
C-allele	326	345	0.906	0.908		354	317	0.885	0.932	
T-allele	34	35	0.094	0.092		46	23	0.115	0.068	
*P_HWE*			0.1617	0.162		0.0661		0.14013	0.3441	
GPX1(rs1800668)					0.1401					0.3982
CC	205	207	0.804	0.881		187	181	0.379	0.823	
CT	49	22	0.192	0.094		48	33	0.2	0.15	
TT	1	6	0.004	0.026		5	6	0.021	0.027	
C-allele	459	436	0.9	0.928		422	395	0.879	0.898	
T-allele	51	34	0.1	0.072		58	45	0.121	0.102	
*P_HWE*			0.2808	0				0.3634	0.0066	
GPX1(rs3811699)					0.8763					0.14013
TT	200	212	0.84	0.841		205	207	0.804	0.881	
CT	34	37	0.143	0.147		49	22	0.192	0.094	
CC	4	3	0.017	0.012		1	6	0.004	0.026	
T-allele	434	461	0.912	0.915		459	436	0.9	0.928	
C-allele	42	43	0.088	0.085		51	34	0.1	0.072	
*P_HWE*			0.08364	0.3467				0.2808	0	
DIO2(rs225014)					0.6574					0.4836
TT	170	175	0.73	0.742		70	81	0.348	0.382	
CT	56	56	0.24	0.237		105	106	0.522	0.5	
CC	7	5	0.03	0.021		26	25	0.129	0.118	
T-allele	396	406	0.85	0.86		245	268	0.609	0.632	
C-allele	70	66	0.15	0.14		157	158	0.391	0.368	
*P_HWE*			0.3712	0.8347				0.16748	0.2748	
TrxR2(rs5748469)					0.4689					0.6574
AA	136	209	0.716	0.749		170	175	0.73	0.742	
AC	49	63	0.258	0.226		56	56	0.24	0.237	
CC	5	7	0.026	0.025		7	5	0.03	0.021	
A-allele	321	481	0.845	0.862		396	406	0.85	0.86	
C-allele	59	77	0.155	0.138		70	66	0.15	0.14	
*P_HWE*			0.8164	0.3958				0.3712	0.8347	

**Table 6:** Genotype and allele frequencies of polymorphisms across selenoprotein genes by serum selenium concentration in Tibetan population.

SNPs	Controls	KBD	Controls	KBD	Trend	Higher group	Lower group	Higher group	Lower group	Trend
	number	number	Freq.	Freq.	p-value	number	number	Freq.	Freq.	p-value
GPX1(rs1050450)					0.932					0.2849
CC	147	155	0.812	0.816		174	125	0.798	0.839	
CT	34	35	0.188	0.184		44	24	0.202	0.161	
TT	0	0	0	0		0	0	0	0	
C-allele	328	345	0.906	0.908		392	274	0.899	0.919	
T-allele	34	35	0.094	0.092		44	24	0.101	0.081	
*P_HWE*			0.1631	0.162				0.2849	0.0975	
GPX1(rs1800668)					0.701					0.924
CC	182	197	0.809	0.804		173	206	0.797	0.811	
CT	39	41	0.173	0.167		41	40	0.189	0.157	
TT	4	7	0.018	0.029		3	8	0.014	0.031	
C-allele	403	435	0.896	0.888		387	452	0.892	0.89	
T-allele	47	55	0.104	0.112		47	56	0.108	0.11	
*P_HWE*			0.2706	0.0121				0.749	0.002	
GPX1(rs3811699)					0.887					0.8969
TT	201	212	0.841	0.841		185	228	0.833	0.848	
CT	34	37	0.142	0.147		35	36	0.158	0.134	
CC	4	3	0.017	0.012		2	5	0.009	0.019	
T-allele	436	461	0.912	0.915		405	492	0.912	0.914	
C-allele	42	43	0.088	0.085		39	46	0.088	0.086	
*P_HWE*			0.082	0.347				0.018	0.8099	
DIO2(rs225014)					0.19					0.6357
TT	57	94	0.425	0.337		63	88	0.389	0.351	
CT	61	150	0.455	0.538		72	139	0.444	0.554	
CC	16	35	0.119	0.125		27	24	0.167	0.096	
T-allele	175	338	0.653	0.606		198	315	0.611	0.627	
C-allele	93	220	0.347	0.394		126	502	0.389	0.373	
*P_HWE*			0.9585	0.0359				0.0035	0.4085	
TrxR2(rs5748469)					0.461					0.6092
AA	136	209	0.716	0.749		154	191	0.748	0.726	
AC	49	63	0.258	0.226		47	65	0.228	0.247	
CC	5	7	0.026	0.025		5	7	0.024	0.027	
A-allele	321	481	0.845	0.862		355	447	0.862	0.85	
C-allele	59	77	0.155	0.138		57	79	0.138	0.15	
*P_HWE*			0.8164	0.3958				0.5367	0.606	

**Table 7:** Statistical Power Calculations for a Paired t Test and Their Effect on Desired Sample Size.

	iodine(case/control)	selenium(case/control)	iodine(high/low)	selenium(high/low)
α	0.05	0.05	0.05	0.05
1-β	0.8	0.8	0.8	0.8
n	90	1337	3	56

α, (alpha)the threshold value below which statistical significance will be declared.

1-β, (one minus beta) the statistical power.

n, the sample size.

### Comparison of the Results of the Genotype Analysis Based on the Higher and Lower Serum Iodine or Selenium Concentration Groups

To further investigate the association of the five SNPs with the serum iodine or selenium concentration, we analyzed the genotype of those in the higher group and lower group of serum iodine or selenium concentration in the Tibetan population (subjects combined the patients and the controls). The mean serum iodine concentration of the combined samples including both cases and controls was 37.33 μg/L. The mean serum iodine concentration of the combined samples including both cases and controls was 37.33 μg/L. The mean serum iodine concentration in the lower group was 24.35 μg/L and 51.64 μg/L in the higher group. The mean serum selenium concentration was 28.65 μg/L. In the lower group, it was 18.18 μg/L, and in the higher group it was 44.40μg/L. The results are presented in the right column of Table 5 and Table 6 for iodine and selenium, respectively. The power calculation results are shown in Table 7. The *GPX1* gene (rs1050450) showed a potential significant association with the iodine concentration, with trend *p* value of 0.02726. However, rs1050450 was not associated with the selenium concentration (trend *p* value  = 0.2849). These might be because the sample size is two less than the power calculated number size 1337 (Table 7). Other SNPs showed no significant association with either iodine or the selenium concentration.

## Discussion

KBD is believed to be a complex disease involving genetic factors, as well as environmental factors such as selenium and iodine deficiency [1]. Based on a candidate gene approach, Xiong *et al*. reported that SNP rs1050450 in the selenoprotein *Gpx1* gene was significantly associated with KBD in a Han Chinese population [21], potentially linked this genetic variant to selenium and iodine deficiency in patients. In China, Tibetans who live in the plateau region are one of the most susceptible populations to KBD. Thus, in this study, we examined the genotype of five selenoprotein SNPs including rs1050450, rs1800668, and rs3811699 in the *Gpx1*gene, rs225014 in the *Dio2* gene, and rs5748569 in the *TrxR2* gene in KBD patients and controls in a Tibetan population. Our results provided no support for any one of the five SNPs being significantly associated with KBD in the Tibetan population. However, haplotype analysis of SNPs rs1050450, rs1800668 and rs3811699 in *GPX1* gene showed a significant association of KBD. Our previous study indicated that the concentrations of serum selenium and iodine in KBD patients were significantly lower than that of controls living in the same village [23], suggesting that genetic variants may affect selenium and/or iodine metabolism. In this study, by haplotype analysis, we found haplotypes TCC, TTC and TTT generated from three SNPs rs1050450, rs1800668, and rs3811699 of *gpx1* gene proved to be significantly associated with KBD and play a protective role from the disease. This is consistent with the fact that supplement of selenium is beneficial for KBD children [13].

In this study, for the first time, we observed that the *GPX1* gene rs1050450 is significantly associated with the serum iodine concentration (*P* trend  = 0.027). The *GPX1* gene encodes a member of the glutathione peroxidase family; SNP rs1050450 in this gene is polymorphic at codon 200, resulting in either a proline or a leucine at that position. Previous studies have suggested that the GPX1-200Leu variant has about 10% lower GPX activity than the wild-type enzyme [24] and that the frequency of the Leu allele is strongly associated with the risk of cancer, such as lung cancer, breast cancer, meningioma, and prostate cancer [25]–[29]. In the present study, we observed that the frequency of the Leu allele of rs1050450 is associated with a relatively higher iodine concentration.

**Figure 2:**
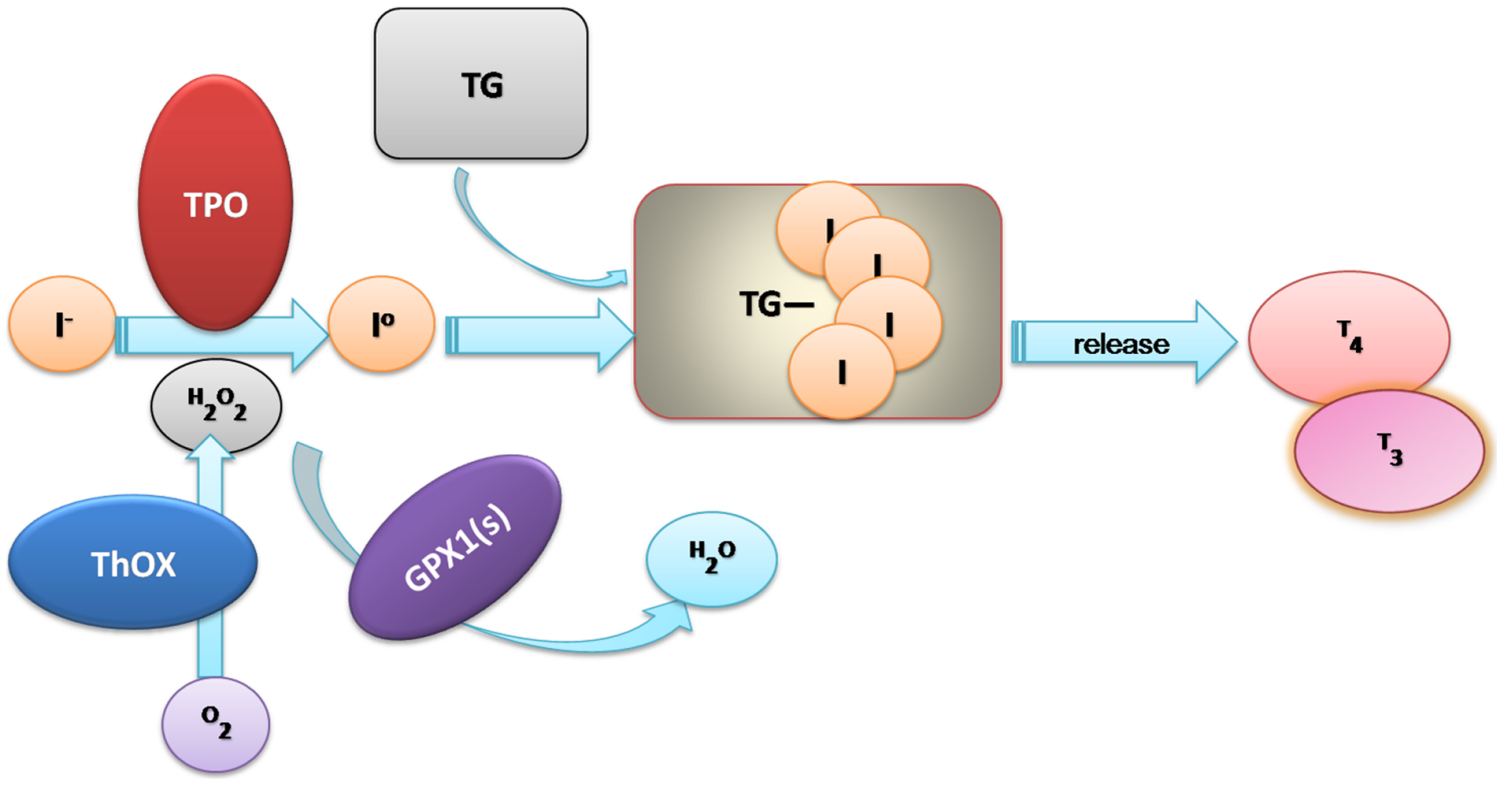
Iodine and GPXs involved in Thyroid Hormones Biosynthesis. The serum sodium iodide is transported into the thyrocyte and then iodine is incorporated into the thyroglobulin molecule (Tg) in a reaction catalyzed by the hemoprotein thyroid peroxidase (TPO). In this reaction, H_2_O_2_ generated by the NADPH-dependent thyroxidase (ThOx) is required as substrate by TPO for the iodination and coupling of tyrosyl residues in Tg. Then, thyroid hormones triiodothyronine (T3) and tetraiodothyronine (T4) are released into the bloodstream. H_2_O_2_ used in this reaction decreases the amount of H_2_O_2_ that would otherwise be available for damaging oxidation reactions. Selenium-dependent glutathione peroxidase 1 (*GPX 1*) and other GPX s remove H_2_O_2_ from the tissues, also decreasing oxidative damage. (Modified from: J. Köhrle *et al*. Selenium, the thyroid, and the endocrine system. Endocrine Reviews, December 2005, 26(7):944–984; Lyn Patrick, ND. Iodine: deficiency and therapeutic considerations. Alternative Medicine Review, 2008, 13(2):116–127).

Iodine is an essential component of the hormones produced by the thyroid gland. Therefore, iodine is essential for mammalian life [30]. In mammals, after iodine is absorbed and distributed in the serum, most of it will be transported by the sodium/iodide symporter (NIS) symporter into the thyrocyte where iodine is oxidated in a reaction catalyzed by the hemoprotein thyroid peroxidase (TPO) and then incorporated into the thyroglobulin molecule (Tg-I) [30]–[32] (Fig. 2). In this reaction, H2O2 is generated by the NADPH-dependent thyroxidase (ThOx) and is required as a substrate by TPO for the iodination in Tg. The thyroid hormones triiodothyronine (T3) and tetraiodothyronine (T4) are then released into the bloodstream. When the iodine supply is sufficient, this H2O2 generation is the limiting step for thyroid hormone synthesis. Therefore, if the KM of TPO for H2O2 is very high, the higher amounts of H2O2 are produced than be consumed by the iodination process [34], the more likely potentially exposing the thyroid gland to free radical damage can be produced. Thus, to prevent organ damage, H2O2, H2O2 should be reduced to H2O immediately after the iodination process. Several selenoproteins participate in the protection of thyrocytes and the prevention of damage to the thyroid gland of H2O2 by catalyzing glutathione and H2O2 to glutathione disulfide and H2O [30]. Selenium-dependent *GPX* gene is one of the first and most important antioxidant enzymes identified in humans and one of only a few proteins known in higher vertebrates to contain selenocysteine. *GPX1* can remove H2O2 from many tissues and cells to decrease oxidative damage. By searching human genes' expression data, we observed that *GPX1* is very highly expressed in thyroid tissue (http://www.genecards.org/cgi-bin/carddisp.pl?gene=GPX1&search=GPX1). This suggests that this gene is an important H2O2 remover in the thyroid gland. When iodine is deficient, the thyroid would increase the generation of H2O2 to maintain the balance of thyroid hormone synthesis. Therefore, the gland requires a higher level of GPX activity to clear the H2O2 and prevent H2O2-induced damage. In contrast, when iodine is sufficient, less H2O2 and GPX are needed to maintain the hormone balance. Thus, we suspect that the iodine concentration and *GPX1* genetic variant might have undergone adaptive evolution in the distant past.

The biosynthesis of selenoproteins is highly regulated by its upstream effectors [33], [34] and by the supply of selenium, which acts as a substrate for the first step in the biosynthesis of Sec-containing proteins. Yet we found no association between the genetic variant Sec-containing coding gene *GPX1* and the selenium concentration in this study. This might because the variant of rs1050450 does not affect the yield of *GPX*, although it does affect the activity of *GPX1*. As the selenium concentration can affect the mole yield of Sec-containing GPX, it can, therefore, influence the activity of the enzymes. Thus, iodine deficiency increases H2O2 generation, whereas selenium deficiency decreases H2O2 disposal [30]. This results in oxidative cell damage causing chronic inflammation or autoimmune diseases. This linkage may explain why selenium and iodine deficiency are associated with many diseases, such as KBD and other impaired immune function–related disease [14], [32].
